# Protective function of DJ-1/PARK7 in lipopolysaccharide and ventilator-induced acute lung injury

**DOI:** 10.1016/j.redox.2020.101796

**Published:** 2020-11-17

**Authors:** Hajera Amatullah, Tatiana Maron-Gutierrez, Yuexin Shan, Sahil Gupta, James N. Tsoporis, Amir K. Varkouhi, Ana Paula Teixeira Monteiro, Xiaolin He, Jun Yin, John C. Marshall, Patricia R.M. Rocco, Haibo Zhang, Wolfgang M. Kuebler, Claudia C. dos Santos

**Affiliations:** a Keenan Research Center of St. Michael's Hospital, Unity Health Toronto, 30 Bond Street, Toronto, ON, Canada; b Department of Physiology, Faculty of Medicine, University of Toronto, Toronto, ON, Canada; c Laboratory of Pulmonary Investigation, Carlos Chagas Filho Institute of Biophysics, Federal University of Rio de Janeiro, RJ, Brazil; d Institute of Medical Sciences, University of Toronto, Toronto, ON, Canada; e Department of Thoracic Surgery, Zhongshan Hospital of Fudan University, Shanghai, 200032, China

**Keywords:** DJ-1, PARK7, Acute lung injury, VILI

## Abstract

Oxidative stress is considered one of the early underlying contributors of acute lung injury (ALI) and ventilator-induced lung injury (VILI). DJ-1, also known as PARK7, has a well-established role as an antioxidant. We have previously shown maintaining oxidative balance via the ATF3-Nrf2 axis was important in protection from ALI. Here, we exclusively characterize the role of DJ-1 in sterile LPS-induced ALI and VILI. DJ-1 protein expression was increased after LPS treatment in human epithelial and endothelial cell lines and lungs of wild-type mice. DJ-1 deficient mice exhibited greater susceptibility to LPS-induced acute lung injury as demonstrated by increased cellular infiltration, augmented levels of pulmonary cytokines, enhanced ROS levels and oxidized by-products, increased pulmonary edema and cell death. In a two-hit model of LPS and mechanical ventilation (MV), DJ-1 deficient mice displayed enhanced susceptibility to inflammation and lung injury. Collectively, these results identify DJ-1 as a negative regulator of ROS and inflammation, and suggest its expression protects from sterile lung injury driven by high oxidative stress.

## Highlights

• Oxidative stress contributes to acute- and ventilator-induced lung injury.
• DJ-1 deficiency increased susceptibility to LPS- and ventilator-induced lung injury.
• DJ-1 as antioxidant protects from sterile lung injury driven by high oxidative stress.

## Graphical abstract

**Figure:**
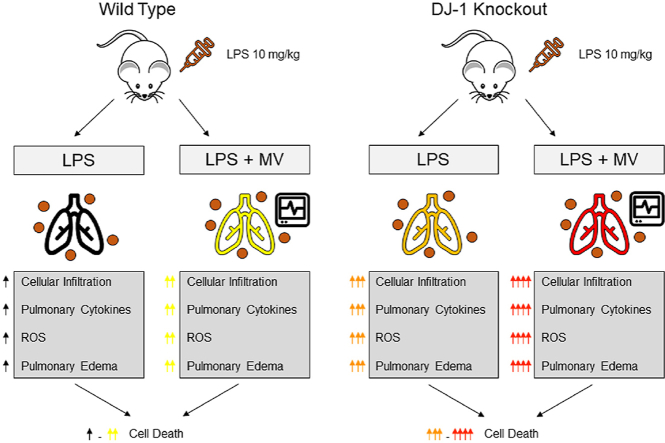

## 1 Introduction

Acute Respiratory Distress Syndrome (ARDS) is a critical complication of systemic or pulmonary infectious or inflammatory processes, and significantly contributes to morbidity and mortality in critically ill patients [1,2]. Infectious etiologies, such as sepsis and pneumonia, are leading causes of ARDS [2,3]. Other non-infectious insults such as hemorrhagic shock, acid aspiration, and systemic inflammatory conditions such as pancreatitis and endotoxemia, can also lead to ARDS [[2], [3], [4], [5], [6], [7], [8]]. The pathological features of ALI/ARDS include inflammatory cell infiltration, increased levels of oxidative stress, pro-inflammatory mediator production, disrupted vascular permeability, and apoptosis [1,9,10]. Both alveolar epithelial and capillary endothelial cell injury and/or death have been shown to contribute to the pathogenesis of ARDS [2,11]. The physiological hallmark of ARDS is alteration of the alveolar–capillary membrane barrier (i.e., increased pulmonary vascular leakage), leading to pulmonary edema, in which protein-rich fluids flood the alveolar spaces, impair gas exchange, and culminate in respiratory failure [1]. Protective mechanical ventilation strategies can lead to further exacerbation of lung injury caused by over-distention and repetitive mechanical stretch stress associated with ventilator-induced lung injury (VILI) [[12], [13], [14]]. Pharmacological approaches to alleviate ARDS/VILI do not currently exist but may be rapidly emerging [15].

Oxidative stress-induced damage is considered to be a fundamental mechanism of injury in ALI and VILI [[16], [17], [18], [19]]. An imbalance in increased levels of oxidants and impaired induction of antioxidants has been documented in experimental ALI models and in ARDS patients [16,[20], [21], [22], [23], [24]]. Plasma concentration of lipid peroxidation products and oxidatively modified proteins are increased in ARDS patients [22,[25], [26], [27]]. Moreover, plasma levels of endogenous antioxidants are decreased [28,29]. Cumulative evidence suggests that in severe injury, persistent oxidative stress overwhelms the endogenous antioxidant capacity. This imbalance has been shown to lead to epithelial and endothelial cell injury and death [30].

Nuclear factor (erythroid-derived 2)-like 2 (NFE2L2 or Nrf2), is a master transcription factor that regulates cellular antioxidant responses [19,31]. Upon exposure to oxidative stress, xenobiotics, or electrophilic compounds, Nrf2 dissociates from Kelch-Like ECH Associated Protein 1 (Keap1) and locates to the nucleus where it binds to the antioxidant response element in the 5′UTR of antioxidant genes including NAD(P)H quinone oxidoreductase 1 (NQO-1), Heme oxygenase 1 (HMOX-1), and Glutathione Peroxidase 1 (GPX-1) [31]. This canonical pathway plays a critical role in experimental models of ALI [[32], [33], [34]]. Non-canonical mechanisms of Nrf2 regulation and stability may also contribute to ALI. Our lab has established a role for activating transcription factor 3 (ATF3) and Parkinson Disease (Autosomal Recessive, Early Onset) 7 or DJ-1 in the regulation of Nrf2 [35,36]. Absence of ATF3 confers marked susceptibility to ALI and VILI by accelerating Nrf2 degradation [35]. Loss of Nrf2 in ATF3 deficient animals results in increased oxidative stress, inactivation of DJ-1/PARK7 and loss of its antioxidant function leading to increased Keap1 mediated Nrf2 ubiquitination [36]. DJ-1 is a ROS scavenger. Its role as a positive regulator of Nrf2 stability and expression has been characterized in H157 non-small-cell lung carcinoma cells, Beas-2b cells and chronic obstructive pulmonary disease patients [37,38]. Here we extend our previous studies by demonstrating the role of DJ-1 in a sterile model of ALI and VILI utilizing gene deficient animals in an intra-tracheal lipopolysaccharide (LPS)-induced acute ALI model as well as a ‘two-hit’ model of VILI.

## 2 Results

### 2.1 DJ-1 expression is increased in epithelial and endothelial pulmonary cells following oxidative and inflammatory stimulation

**Fig. 1:**
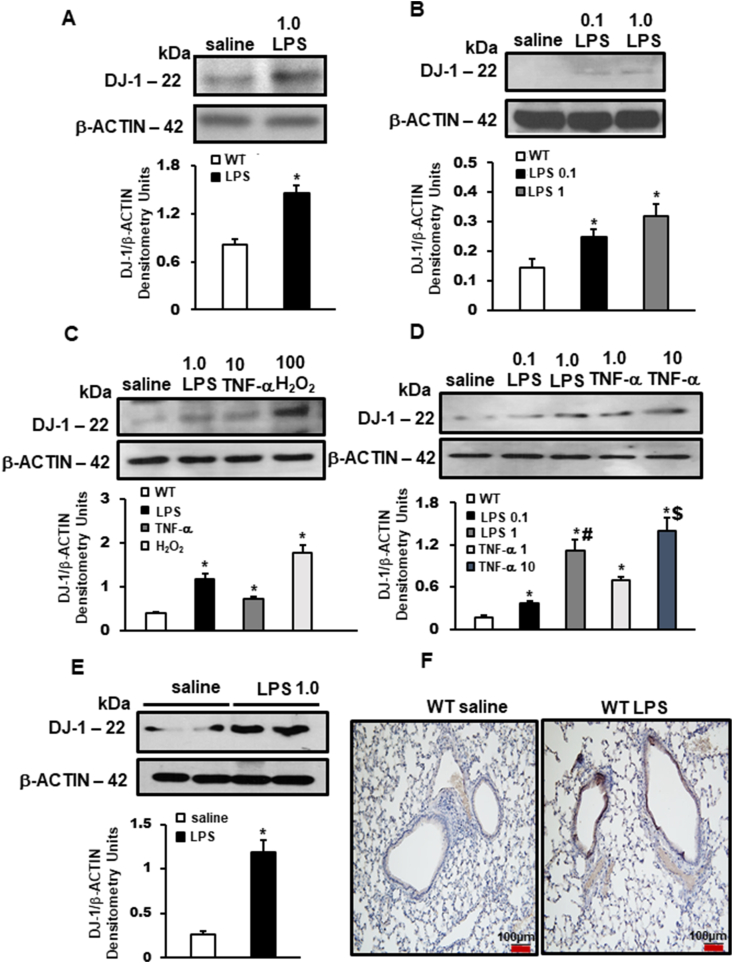
**Increased DJ-1 expression in human lung cells and mice following oxidative and inflammatory stimuli.** Doses: LPS - μg/mL, TNF-α - ng/mL, H_2_O_2_ - μM. Representative western blots showing increased DJ-1 protein expression in **A)** A549 cells (n = 2 independent experiments), **B)** HPMEC cells (n = 2 independent experiments), **C)** Beas-2b cells with different stimuli (n = 3 independent experiments), and **D)** Beas-2b cells with different doses (n = 3 independent experiments), following stimulation normalized to β-ACTIN protein expression. **E)** Representative Western blot and showing increased DJ-1 protein expression in whole lung lysates, following LPS stimulation normalized to β-ACTIN protein expression. Densitometry measurements (protein/β-actin) are mean + SEM. *<0.05 vs. saline, #p < 0.05 vs. LPS 0.1, $p < 0.05 vs. TNF-α 1. **F)** Representative immunohistochemistry images of DJ-1 expression (DAB brown staining) in lungs following 24 h of LPS instillation.

DJ-1 protein expression was increased in human alveolar basal epithelial cells (A549), human pulmonary microvascular endothelial cells (HPMEC), and distal bronchial small airway epithelial cells (BEAS2b) following exposure to LPS (Fig. 1A–C). Exposure of BEAS2b cells to tumor necrosis factor (TNF-α) or hydrogen peroxide also induces increased DJ-1 expression (Fig. 1C and D). In separate experiments, the expression of DJ-1 was induced in a dose dependent manner by both LPS and TNF-α (Fig. 1D).

### 2.2 Effect of DJ-1 in LPS-induced acute lung injury (ALI)

LPS intratracheal instillation (10 mg/kg) for 24 h increased the expression of DJ-1 in lungs from wild-type (WT) mice compared with saline controls (Fig. 1E). Immunohistochemical staining for DJ-1 in WT lungs post LPS instillation reveal that DJ-1 is expressed in both infiltrating myeloid cells as well as parenchymal cells, with the bulk of the increase in expression localizing to airway epithelial and peri-bronchial cells (Fig. 1F).

**Fig. 2:**
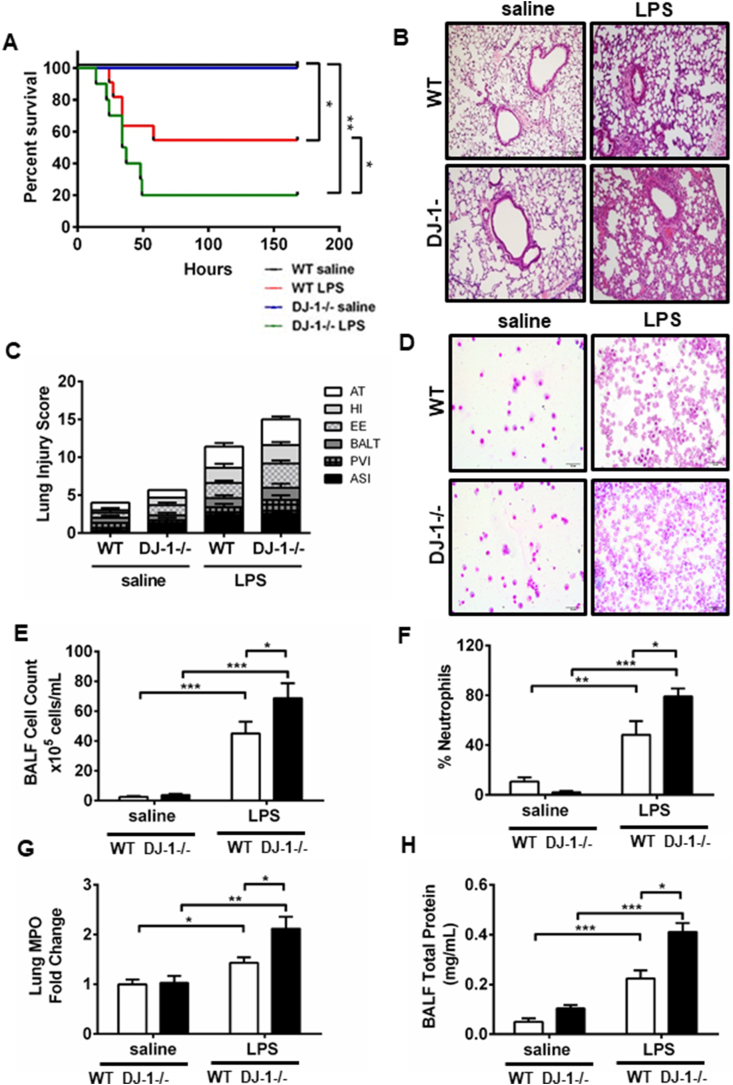
**Absence of DJ-1 exacerbates LPS-induced acute lung injury. A)** Percent survival of wildtype (WT) and DJ-1−/− mice at 7 days after LPS instillation compared with saline (SAL) controls. (saline n = 5, LPS n = 10–11, *p < 0.05 **p < 0.01, Log-rank/Mantel-Cox analysis) **B)** Representative H&E stained images of lungs 24 h post-instillation of SAL or LPS. **C)** Lung Injury Scores (LIS) (n = 3–5 animals) as characterized previously using alveolar septum thickening (AT), hemorrhagic infiltration (HI), exudative edema (EE), bronchus-associated lymphoid tissue aggregates (BALT), perivascular inflammatory infiltrates (PVI), and alveolar septal infiltration (ASI). **D)** Representative H & E stained images of Bronchoalveolar lavage fluid (BALF) cyto-spins from WT and DJ-1−/− mice exposed to saline or LPS treatment. BALF **E)** total cell count (n = 8–13), and **F)** percent neutrophils (n = 8–13) in WT and DJ-1−/− mice at 24 h after saline and LPS treatment. **G)** Myeloperoxidase levels in lung tissue lysates with saline or LPS treatment (n = 7–14). **H)** BALF total protein (n = 7–14) at 24 h after saline or LPS treatment. Data are presented as means ± SEM (*p < 0.05, **p < 0.01, ***p < 0.001, two-way ANOVA analysis).

To determine the role of DJ-1 in a model of sterile injury, WT and DJ-1 deficient (DJ-1−/−) mice were randomized to LPS (10 mg/kg) or equal volume of saline and observed for 7 days. DJ-1−/− mice demonstrated susceptibllity to LPS-induced mortality compared with WT mice (Fig. 2A). Increased mortality of DJ-1−/− mice was further associated with impaired pulmonary mechanics as evidenced by a trend towards worsening of the pressure-volume relationship (Supplementary Fig 1A). Levels of atelectasis, as determined by the area of the pressure volume loops (Table 1), increased modestly with LPS treatment but did not show a significant difference between genotypes. Quasistatic compliance (Cst) was decreased and elastance was increased in DJ-1−/− animals compared with WT animals following LPS treatment. In a single compartment model analysis, total dynamic resistance (Rrs) significantly increased in the DJ-1−/− mice stimulated with LPS (Supplementary Fig 1B); conversely, pulmonary compliance, a hallmark feature of ARDS, was further reduced in the absence of DJ-1 (Supplementary Fig 1C). Following partitioned respiratory mechanics assessment, central airways Newtonian resistance (R_N_) as well as peripheral tissue dampening (H), and tissue elastance (E) in LPS treated mice were also augmented in the DJ-1−/− compared to WT mice (Table 1). Collectively, these results suggest that DJ-1−/− leads to additional deterioration in lung functional parameters following endotoxin challenge.

**Table 1:** Effects of DJ-1 loss on respiratory mechanics following 24 h of LPS instillation.

	WT SAL	DJ-1−/− SAL	WT LPS	DJ-1−/− LPS
PV Loops				
**Cst (mL/cm**H_**2**_**O)**	0.086 ± 0.010	0.073 ± 0.006 $	0.070 ± 0.004 *	0.059 ± 0.004 *$
**Est (cmH**_**2**_**O/mL)**	12.100 ± 1.522	14.044 ± 1.048	14.430 ± 0.882	17.718 ± 1.488 *$
**A**	0.865 ± 0.043	0.747 ± 0.048	0.737 ± 0.031	0.700 ± 0.034
**B**	1.244 ± 0.166	1.018 ± 0.075	1.065 ± 0.059	0.910 ± 0.060
**K**	0.138 ± 0.013	0.130 ± 0.004	0.130 ± 0.007	0.131 ± 0.004
**Area**	2.490 ± 0.192	2.032 ± 0.104	2.901 ± 0.217*	3.339 ± 0.194*
***Partitioned Mechanics***				
**Rn (cmH**_**2**_**O.s/mL)**	0.221 ± 0.018	0.244 ± 0.016	0.258 ± 0.019	0.340 ± 0.031*$
**G (cmH**_**2**_**O/mL)**	5.033 ± 0.692	4.649 ± 0.739	5.435 ± 0.341	6.984 ± 0.637 *$
**H (cmH**_**2**_**O/mL)**	23.509 ± 0.779	23.055 ± 0.481	26.315 ± 1.371	34.134 ± 3.402 *$
***Lung Volume***				
**IC/weight (mL/kg)**	48.748 ± 1.673	41.576 ± 1.216 $	40.403 ± 2.352 *	42.717 ± 1.702

* denotes p < 0.05 statistically significant compared to saline; $ denotes p < 0.05 compared to the other genotype; SAL – saline; Cst – quasistaic compliance; Est – quasistatic elastance; A – estimate of inspiratory capacity; B - estimate of the difference between the volume at total lung capacity and the predicted volume at zero pressure; K - curvature of the upper portion of the deflation limb of the PV curve; Area - area enclosed by the pressure volume loop provides an estimate of the amount of atelectasis; Rn – Newtonian resistance of the central or conducting airways; G – Tissue damping represents resistance and energy dissipation in the alveoli; H – tissue elastance reflects energy conservation in the alveoli; IC/wt – Inspiratory capacity normalized to body weight.

### 2.3 DJ-1 protects mice from endotoxin-induced inflammation and alveolar capillary membrane dysfunction

The pathological hallmark of ALI in humans is diffuse alveolar damage (DAD). Lung histology and morphology (Fig. 2B) showed increased lung injury in the DJ-1−/− compared with WT mice, as characterized by denudation of the alveolar membrane, increased cellular infiltration, edema formation, and hyaline membrane deposition – all elements of the lung injury score (LIS, Fig. 2C). BALF collected 24 h after LPS instillation also showed a significant increase in total cell and neutrophil recruitment in DJ-1−/− compared with WT mice (Fig. 2D, E, 2F). This was concurrent with increased myeloperoxidase (MPO) levels in lung tissue lysates of LPS challenged DJ-1−/− compared to wild-type mice (Fig. 2G).

Alveolar permeability was also increased in the DJ-1 −/− mice as assessed by gross extravasation of Evans Blue Dye (EBD, Supplementary Fig 1D). BALF total protein was significantly increased in the BALF from DJ-1−/− versus corresponding WT controls (Fig. 2H). We did not see a difference between genotype in BALF IgM levels following LPS treatment (Supplementary Fig 1E). Taken together, these data demonstrate that DJ-1 deficiency increases susceptibility to LPS-induced inflammation and impairment of alveolar capillary barrier function.

### 2.4 DJ-1 negatively regulates inflammation following LPS stimulation

**Fig. 3:**
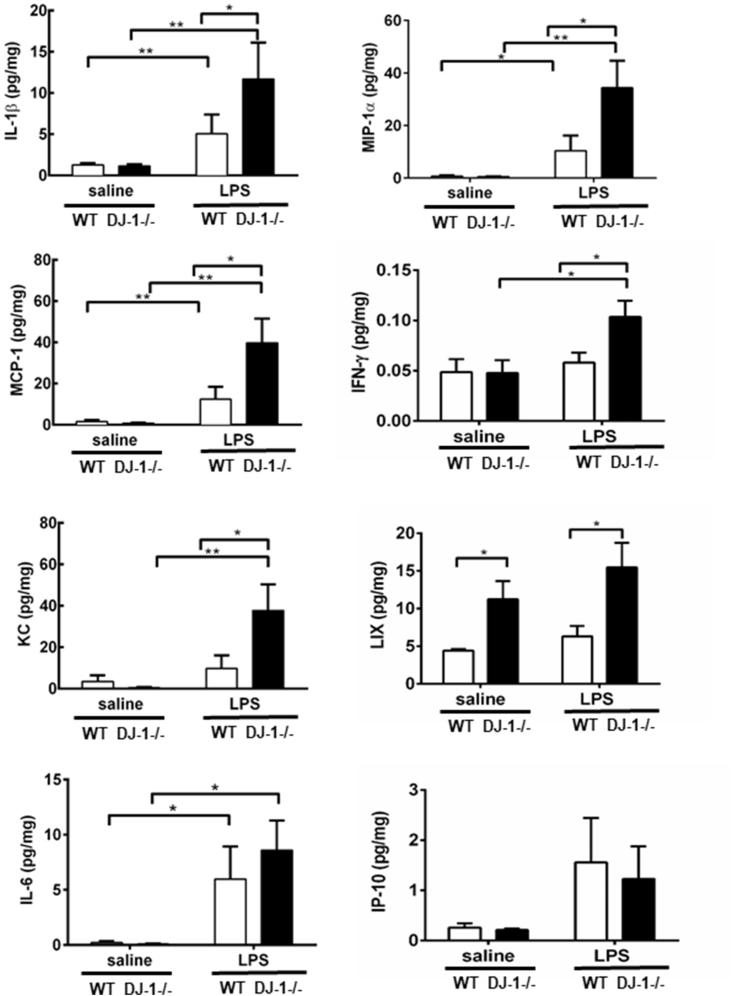
**DJ-1 deficiency enhances lung inflammation following LPS instillation.** Levels of inflammatory mediators in lung homogenates and serum from WT or DJ-1−/− mice 24 post LPS instillation. Mediators profiled: interleukin-1 beta (IL-1β), macrophage inflammatory protein 1 alpha (MIP-1α/CCL3), monocyte chemoattractant protein-1 (MCP-1/CCL2), interferon gamma (IFN-γ), keratinocyte chemoattractant (KC/CXCL1), LIX(CXCL5), interleukin-6 (IL-6) and interferon gamma-induced protein 10 (IP-10/CXCL10. Data are presented as means ± SEM (n = 6–8 per group, *p < 0.05, **p < 0.01, two-way ANOVA).

Increased cell infiltration was observed with a significant increase in lung tissue levels of pro-inflammatory cytokines: IL-1β, MIP-1-α, MCP-1, KC, IL-6 and LIX (Fig. 3). Interestingly, LPS did not affect IP-10 expression. Taken together, our data demonstrates that in comparison to the WT mice, DJ-1−/− mice have enhanced cellular infiltration and inflammation, highlighting DJ-1's role as a negative regulator of inflammation in sterile lung injury.

### 2.5 DJ-1 reduces ROS levels and promotes an antioxidant response in ALI

**Fig. 4:**
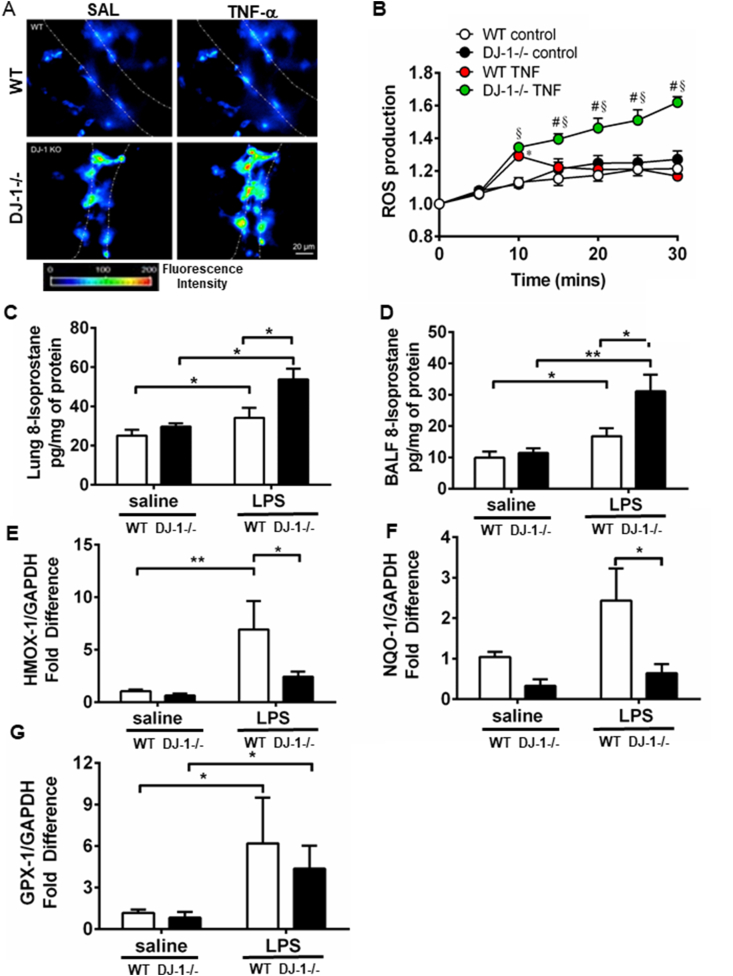
**Loss of DJ-1 aggravates pulmonary oxidative stress following LPS stimulation. A)** Representative images of H_2_DCFDA-loaded endothelial cells in micro-vessels of the isolated perfused mouse lung showing imaging of endothelial DCF with increasing fluorescence intensity in the DJ-1−/− **B)** and quantitative analysis of DCF fluorescence relative to baseline showing increased ROS production in response to TNF-α infusion (1000 U/mL) in DJ-1−/− compared to WT lungs. Data are presented as means ± SEM (n = 3–5 mice per group, *p < 0.05 compared to WT saline and #p < 0.05 compared to WT TNF treated). 8-Isoprostane levels in **C)** lung lysates and **D)** BALF in WT and DJ-1−/− animals following LPS instillation. Bar graphs represent means ± SEM (n = 7–9 per group, *p ≤ 0.05, **p ≤ 0.01, two-way ANOVA). Real-time PCR analysis of **E)** HMOX-1, **F)** NQO-1 and **G)** GPX-1 mRNA levels in WT and DJ-1−/− lungs 24 h following LPS instillation. Bar graphs represent means ± SEM (n = 6–8 per group, *p ≤ 0.05, **p ≤ 0.01, two-way ANOVA).

Following TNF-α stimulation, ROS production is markedly increased in DJ-1−/− compared to WT lungs (Fig. 4A and B). Iso-prostanoids are prostaglandin-like compounds formed from the free radical-catalyzed peroxidation of essential fatty acids and are associated with increased oxidative stress. 8-isoprostane levels were increased in lung lysates and BALF in LPS challenged DJ-1 deficient mice (Fig. 4C and D). Compared to WT, DJ-1−/− mice had decreased Nrf2 protein and mRNA expression as well as decreased Nrf2 activity as demonstrated by decreased expression of Nrf2-dependent genes, HMOX-1, NQO-1 and GPX-1 (Fig. 4E, F, G) validating previous findings that DJ-1 is a positive regulator of Nrf2 expression and activity.

### 2.6 DJ-1 protects against increased cell death following endotoxin challenge

**Fig. 5:**
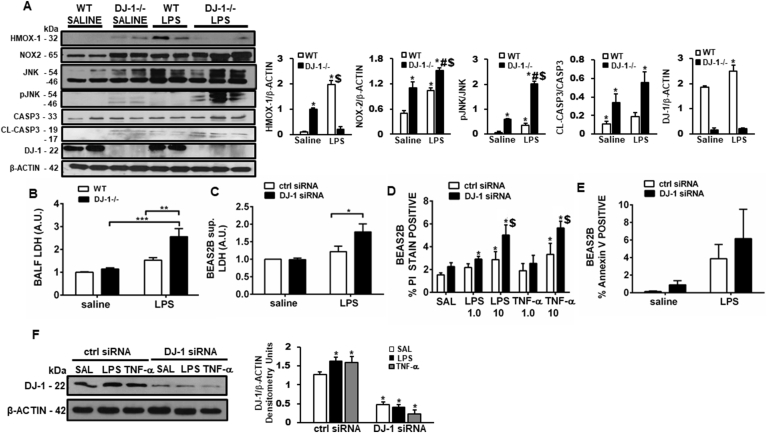
**Loss of DJ-1 leads to oxidative stress-induced enhanced cell death. A)** Western blots showing protein expression of NRF2, HMOX1, NADPH OXIDASE 2 (NOX2), JNK, pJNK, caspase 3 (CASP3), cleaved caspase 3 (CL-CASP3), DJ-1 and B-ACTIN. Densitometry measurements (protein/β-actin) mean + SEM. n = 2–3. *p < 0.05 vs. WT saline, #p < 0.05 vs. WT LPS, $p < 0.05 vs. DJ-1−/− saline. Cell death analysis using Lactate dehydrogenase (LDH) release in **B)** BALF (n = 6–8 animals each) and **C)** supernatants of Beas-2b cells treated with 50 nM control siRNA or DJ-1 siRNA (n = 3 independent experiments. **D)** Propidium Iodide (PI) staining (n = 3 independent experiments) and **E)** Annexin V staining in control and DJ-1 siRNA treated Beas-2b cells (n = 1 independent experiment). *p < 0.05, **p < 0.01, ***p < 0.001, two-way ANOVA.

Increased oxidative stress and inflammation results in increased parenchymal cell apoptosis contributing to the progression of ALI. Since DJ-1 is known to play a role in oxidative stress-induced cell death, we assessed whether cell death contributed to worsening injury in DJ-1−/− mice. In the absence of DJ-1, intratracheal LPS administration results in increased markers of cell death, including NOX2, pJNK and cleaved caspase-3 (Fig. 5A). BALF lactate dehydrogenase (LDH) release, which is an indicator of cell death, was also increased in DJ-1−/− mice (Fig. 5B). These results were recapitulated *in vitro* in a model of LPS-induced cell injury. Increased LDH release in cell supernatants, propidium iodide (PI) staining, Annexin V staining, and cleaved caspase-3 expression were observed in primary bronchoalveolar distal airway epithelial cell Beas-2b transfected with a siRNA against DJ-1 compared to control siRNA (Fig. 5 A, C, D, E & F).

### 2.7 DJ-1 deficiency exacerbates lung injury in a “two-hit” model of VILI

**Fig. 6:**
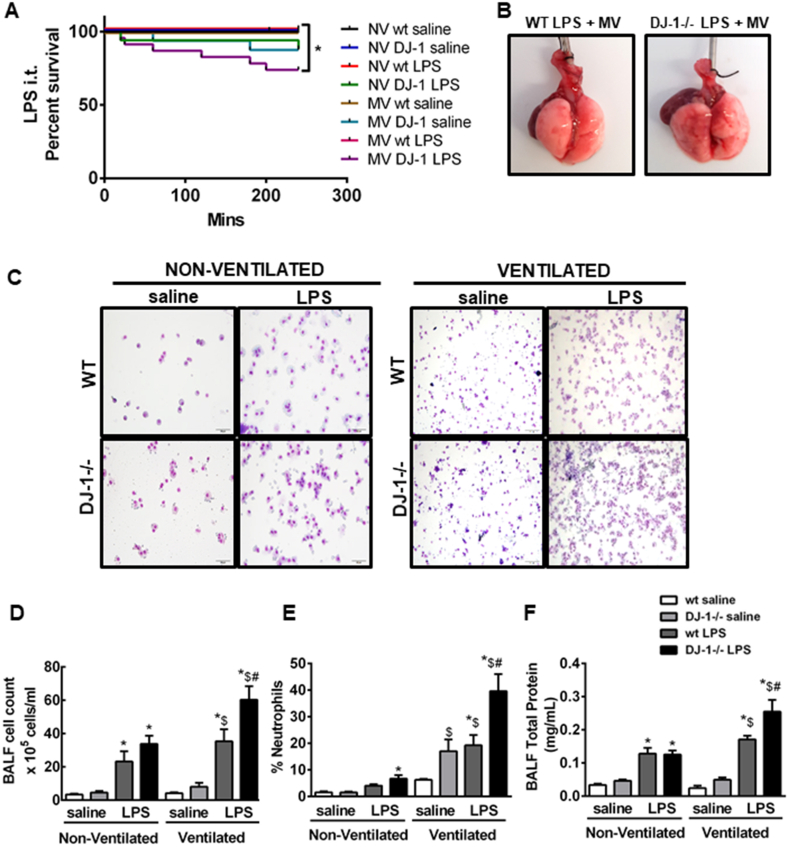
**DJ-1 deficiency exacerbated increased lung injury and impaired function in LPS + MV model. A)** Percent survival of WT and DJ-1−/− mice at 4 h after saline or LPS instillation with or without mechanical ventilation (MV) (*p < 0.05 Log-rank/Mantel-Cox analysis). **B)** Representative images of whole lungs from WT and DJ-1−/− mice following LPS + mechanical ventilation (MV). **C)** Representative images of H & E stained BALF cytospins from WT and DJ-1−/− mice at 4 h after saline or LPS instillation with or without mechanical ventilation (MV). BALF **D)** total cell count, **E)** percent neutrophils and **F)** total protein in WT and DJ-1−/− mice at 4 h post LPS and ventilation. Data are presented as means ± SEM (n = 6–9 saline and 11–17 LPS mice) *p < 0.05 compared with saline control of respective genotype $p < 0.05 compared with non-ventilated LPS group, #p < 0.05 compared with WT LPS ventilated, two-way ANOVA analysis).

To determine whether DJ-1 modulates the innate immune response of the lung during mechanical ventilation, we exposed DJ-1−/− and WT mice to a two-hit model of lung injury. In keeping with our previous results, DJ-1−/− mice showed increased stress and more frequently succumbed to two-hit model injury (Fig. 6A). Morphological and histological examination of lung tissues revealed that DJ-1 deficient animals have increased alveolar hemorrhage, cellular inflammation, and edema formation when mechanical injury (biomechanical) is superimposed on the inflammatory (biochemical) injury (Fig. 6B and Supplementary Fig 2A). Respiratory mechanics were assessed in the 4-h single hit (non-ventilated) model; however, since no changes were observed between the DJ-1−/− and WT mice at this time point the remainder of respiratory mechanic studies were only conducted in the ventilated animals only. Compared to WT, DJ-1 deficient mice had increased dynamic pulmonary resistance and decreased compliance 4 h after MV and LPS instillation (Supplementary Fig 2B and C, Table 2).

**Table 2:** Effects of DJ-1 loss on respiratory mechanics following 4 h of LPS + MV model.

	WT SAL + MV	DJ-1−/− SAL + MV	WT LPS + MV	DJ-1−/− LPS + MV
PV Loops				
**Cst (mL/cmH**_**2**_**O)**	0.077 ± 0.007	0.064 ± 0.006	0.070 ± 0.005	0.059 ± 0.006
**Est (cmH**_**2**_**O/mL)**	12.830 ± 1.021	18.042 ± 2.746 $	13.953 ± 1.131	18.529 ± 3.033
**A**	0.787 ± 0.043	0.697 ± 0.081	0.738 ± 0.060	0.819 ± 0.104
**B**	1.171 ± 0.083	0.942 ± 0.094	1.036 ± 0.075	0.694 ± 0.060 *$
**K**	0.144 ± 0.008	0.131 ± 0.006	0.134 ± 0.006	0.128 ± 0.011
**Area**	3.767 ± 0.487	3.285 ± 0.662	3.746 ± 0.739	3.316 ± 0.935
***Partitioned Mechanics***				
**Rn (cmH**_**2**_**O.s/mL)**	0.300 ± 0.018	0.299 ± 0.027	0.315 ± 0.030	0.296 ± 0.022
**G (cmH**_**2**_**O/mL)**	4.485 ± 0.189	6.671 ± 0.540 $	5.454 ± 0.420 *	6.807 ± 0.752 $
**H (cmH**_**2**_**O/mL)**	26.065 ± 1.088	37.500 ± 5.389 $	28.561 ± 2.077	35.344 ± 4.812
***Lung Volume***				
**IC/weight (mL/kg)**	40.029 ± 1.501	39.604 ± 2.871	37.198 ± 2.551	36.029 ± 3.184

* denotes p < 0.05 statistically significant compared to saline; $ denotes p < 0.05 compared to the other genotype; SAL – saline; Cst – quasistaic compliance; Est – quasistatic elastance; A – estimate of inspiratory capacity; B - estimate of the difference between the volume at total lung capacity and the predicted volume at zero pressure; K - curvature of the upper portion of the deflation limb of the PV curve; Area - area enclosed by the pressure volume loop provides an estimate of the amount of atelectasis; Rn – Newtonian resistance of the central or conducting airways; G – Tissue damping represents resistance and energy dissipation in the alveoli; H – tissue elastance reflects energy conservation in the alveoli; IC/wt – Inspiratory capacity normalized to body weight.

BALF from DJ-1−/− mice consistently showed significant increase in cellular inflammation and neutrophilia compared to WT controls (Fig. 6C, D & E). Moreover, DJ-1−/− mice also had increased total protein in BALF (Fig. 6F), indicating greater loss of alveolar-capillary barrier function.

**Fig. 7:**
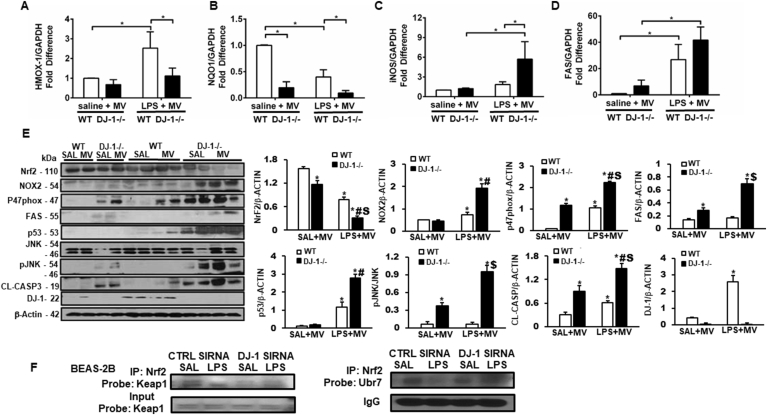
**DJ-1 deficient animals had increased expression of oxidative stress and cell death markers in a “two-hit” model. A)** mRNA Expression of Heme oxygenase-1(HMOX-1), **B)** NADPH quinone oxidoreductase 1 (NQO1), **C)** inducible NOS (iNOS), **D)** and FAS in WT and DJ-1−/− mechanically ventilated (MV) mice with either saline or LPS administration. Data are presented as means ± SEM (n = 6–8 mice, *p < 0.05, two-way ANOVA). **E)** Western blots showing protein expression of Nrf2, NADPH oxidase 2 (NOX2), p47phox, FAS, p53, JNK, pJNK, cleaved caspase-3, DJ-1 and B-ACTIN. Densitometry measurements (protein/β-actin) are mean + SEM. n = 2–4. *p < 0.05 vs. WT saline (SAL) + MV, #p < 0.05 vs. WT LPS + MV, $p < 0.05 vs. DJ-1−/− SAL + MV. **F)** Western blot showing immunoprecipitation with Nrf2 and probing for Keap1 or Ubr7 in Beas-2b cells with control or DJ-1 siRNA (n = 2 independent experiments).

In keeping with our previous results, HMOX-1 and NQO-1 mRNA expression was reduced in DJ-1−/− compared to WT animals (Fig. 7A and B). In addition, iNOS and FAS mRNA expression (Fig. 6C and D) was increased further supporting a protective role for DJ-1 in lung inflammation and cell death. Impaired redox status was also shown with decreased Nrf2 protein expression and increased pro-oxidant Nox2 and p47phox expression (Fig. 7E). While WT animals show minimal injury in the two-hit model, DJ-1−/− mice had increased expression of cell death markers (Fig. 7E) pJNK, Fas and cleaved caspase 3. This occurred even in mice exposed to ventilation alone suggesting DJ-1 modulates lung injury in response to mechanical stress alone.

### 2.8 Loss of DJ-1 enhances Keap1 mediated-Nrf2 degradation

To determine Nrf2 stability following DJ-1 silencing, we pulled down ubiquitin and probed for Nrf2. WT cells have decreased Nrf2 ubiquitination following LPS compared with saline, which is in line with established role of Nrf2 activation following cellular stress (Fig. 7F). Conversely, DJ-1 knockown treated with LPS had enhanced Nrf2 ubiquitination compared with WT LPS-treated cells, suggesting inability to stabilize Nrf2 (Fig. 7F). To show this is dependent on Keap1 function, we pulled down Keap1 in WT and DJ-1 knock down cells and assessed for Nrf2 binding (Fig. 7F). DJ-1 knockown cells had increased Nrf2 binding to Keap1, indicating increased loss of Nrf2 was due to increased Keap1 binding.

## 3 Discussion

Here we report DJ-1 is a negative regulator of oxidative stress and sterile inflammation in ALI/VILI. DJ-1−/− mice were overly sensitive to lung injury induced by non-bacterial models. In our 24 h LPS model, we show that DJ-1 deficiency exacerbates markers of oxidative stress, inflammation, and cell death. In our 4 h model, we similarly found that DJ-1 deficiency enhanced oxidative stress and inflammation, which was synergistically increased by MV. These findings were similarly observed in DJ-1 knocked down Beas2b cells exposed to LPS. The above-mentioned pathogenic processes were accompanied by pulmonary edema and respiratory failure.

In line with its well-established function as a cellular antioxidant, our study showed administration of TNF-α in ex vivo perfused lungs led to greater and sustained ROS production in DJ-1−/− mice. 8-isoprostane levels, a marker of oxidative stress, in the BALF and lung tissues were similarly increased in DJ-1 deficient animals. Moreover, induction of Nrf2 responsive genes (Hmox-1, Nqo1) was reduced in DJ-1−/− mice. We also show DJ-1−/− mice and DJ-1 knocked down cells had increased Nrf2-Keap1 association, implying enhanced Keap1 mediated Nrf2 degradation further proving previously established notion that DJ-1 is a positive regulator of Nrf2 activity. Previously, it was shown that NQO1 and glutamate cysteine ligase modifier (GCLM) was decreased in DJ-1 knockdown H157 and Huh7 cells as knockout primary mouse embryonic fibroblasts (MEFs) [39]. Nrf2 mRNA expression wasn't altered; however, Nrf2 protein was drastically reduced [39].

Oxidative stress-induced cell death has been noted with absence of DJ-1 in various cell types following a multitude of stimuli. Our study similarly showed upregulation of various cell death markers in both models of lung injury and this is observed with cells knocked down with DJ-1 siRNA.

DJ-1 has been shown to be upregulated in human lung cell lines and animal lung tissues following various insults such as cigarette smoke exposure. More importantly, DJ-1 has been shown to be responsive to endotoxin in several studies (although the majority are in neuronal cells and in the setting of neuroinflammation). Our findings of DJ-1 negatively regulating sterile inflammation in the lung following LPS-induced shock is in line with previous studies. Knockdown or loss of DJ-1 in various cell types and tissues led to greater induction of pro-inflammatory cytokines and chemokines. In our study, this was further exacerbated with MV. Importantly, MV alone led to greater oxidative stress and inflammation in DJ-1−/− mice, suggesting for the first time DJ-1 is responsive to mechanical stress. Evidence from cell culture studies in endothelial and epithelial cells have consistently shown increased ROS generation following physiologically relevant cyclic stretch, which was accompanied by either a loss of antioxidant levels (such as GSH), oxidation of GSH and loss of antioxidant (SOD, GPX) activity [[40], [41], [42], [43]]. These results have also been corroborated in *in vivo* animal studies and in humans [44,45]. Based on these findings, we speculate DJ-1 may fall in the latter category where persistent oxidative stress leads to its oxidation and loss of activity. Moreover, ROS generation following cyclic stretch has been associated with increased expression and activity of NADPH oxidase (NOX) and dual oxidase (DUOX) [[46], [47], [48]]. DJ-1−/− mice had increased p47phox and Nox2/gp91phox expression in response to MV irrespective of LPS, suggesting both ventilation and LPS synergistically increase ROS through NADPH oxidase complex.

MV is a life-saving intervention in patients with acute respiratory failure. However, MV itself can contribute to VILI [49]. VILI can exacerbate previously damaged lung or initiate injury in healthy lung, even with low tidal volume protective strategies [[50], [51], [52], [53], [54], [55], [56], [57]]. MV-induced inflammation is partially dependent on TLR4 signaling [51,58,59] and other endogenous danger ligands detection receptors such as NLRP3 [60,61]. The LPS + MV model may offer clinical relevance in recapitulating septic patients that are placed on ventilator in the ICUs. The biophysical stress from MV can serve as a “second-hit” in already compromised hosts, leading to greater pro-inflammatory cytokine induction which can possibly cascade into a cytokine storm and/or systemic inflammation [49,62].

Preclinical *in vivo* models of pulmonary inflammation are commonly used as surrogates for sepsis-induced ALI. Administration of LPS, a gram negative bacterial cell wall component, is often used to recapitulate inflammatory cell influx, pro-inflammatory cytokine production, endothelial dysfunction and pulmonary edema formation [9,10]. LPS increased protein extravasation into the alveolar spaces; was significantly exacerbated in DJ-1−/− mice implying greater loss of vascular integrity. However, we did not observe differences in IgM levels between genotypes. IgM is a large, pentameric molecule detected in BALF with severe vascular permeability. Later time points may reveal differences in IgM levels in the BALF fluid with more injury, which were not statistically significant at 24 h.

Capturing functional changes (i.e., loss of respiratory capacity/respiratory failure) following acute inflammation in small animals has however been less well characterized and inconsistent [[63], [64], [65]]; there are a few studies using FlexiVent (and other forced maneuver systems) that have now shown loss of pulmonary compliance coinciding with LPS-induced inflammatory changes [24,57,66,67]. In our study, we were able to document reduction in pulmonary compliance in our WT animals only in the 24-h model. Reduction in pulmonary compliance in wild-type animals noted in the other studies have generally been observed between 24 and 48 h time points. Earlier time points may not have enough injury in WT animals to cause an effect on respiratory mechanics. Although decreased compliance is generally studied, there is also evidence of increased airway resistance in ALI/ARDS patients (independent of pre-existing respiratory disease) [[68], [69], [70]]. Our model also showed an increased in respiratory resistance following LPS treatment.

We have previously shown that DJ-1−/− mice were protected against cecal ligation and puncture (CLP)-induced polymicrobial sepsis [71]. This is in clear contrast with results from the current study using LPS. Both models revealed DJ-1 is a negative regulator of innate immunity; loss of DJ-1 led to enhanced levels of pro-inflammatory cytokines. In the polymicrobial model of sepsis, however, absence of DJ-1 led to reduced bacterial burden in organs and blood which was associated with increased phagocytosis and killing of bacteria mediated via enhanced NADPH oxidase 2 function [71]. We postulate conflicting data can be explained by the divergent role of DJ-1 in bacterial clearance versus inflammation. In a model without live bacteria, i.e. endotoxin only, the overzealous pro-inflammatory response has no benefit and only leads to collateral tissue damage. This adds to the litany of previous evidence of pro-inflammatory cytokine knockout mice showing variable outcomes depending on the experimental sepsis model utilized. These findings further add to our understanding of distinct host outcomes to infectious versus sterile inflammation insults. Further studies are currently underway to determine how DJ-1 plays a role in “beneficial” (bacterial clearance) versus “harmful” (oxidative stress) cellular processes.

## 4 Conclusions

In conclusion, our data demonstrates that DJ-1 protects against oxidative stress and is a negative regulator of inflammation in ALI/ARDS. Loss of DJ-1 leads to augmented oxidative stress, pro-inflammatory markers, edema formation and impaired lung mechanics. Future studies will investigate how DJ-1 can distinctly sense and regulate diverse signaling pathways following LPS versus live bacterial infection.

## 5 Materials and methods

### 5.1 Cell culture

Human bronchial epithelial cell line (Beas-2b) was maintained in Dulbecco's modified Eagle's Medium (DMEM, Life Technologies Inc) supplemented with 10% FBS and 1% penicillin/streptomycin.

### 5.2 DJ-1 loss- and gain-of-function studies in Beas-2b cells

Beas-2b cells were transfected siRNA respectively against DJ-1 (DJ-1siRNA, loss-of-function), or a control scrambled siRNA, 50 nM (ctrl siRNA, Ambion). Alternatively, BMMs were infected (50 multiplicity of infection, MOI) overnight with recombinant adenovirus overexpressing DJ-1 (Ad-DJ-1, gain-of-function) or a control adenovirus (Ad-Ctrl). Adenovirus vectors were a gift from Dr. David Park (University of Ottawa). After overnight incubation with either siRNA or recombinant adenovirus transfection, cells were treated with 1 μg/mL of LPS (*E. coli* LPS 026:B6 and 055:B5, Sigma-Aldrich, St. Louis) for 24 h. Total protein in cell lysates was estimated by Bradford Assay.

### 5.3 Animals

All protocols were approved by the Animal Care Committee at St. Michael's Hospital (Toronto, ON, CA). Wildtype-type (WT, DJ-1 +/+) C57Bl/6J (Jackson Laboratories) and DJ-1 deficient mice (targeted deletion of DJ-1, DJ-1−/− [72]) on a C57Bl/6J background (20 backcrosses) were housed in a specific pathogen free facility with standard 12 h light/dark cycle with *ad libitum* access to Teklan Global 18% Protein Rodent Diet and RO water. Mice were acclimatized to the animal facilities for at least a week before beginning experiments. Mice were treated humanely in accordance with the guidelines outlined in the Canadian Council of Animal Care.

DJ-1 deficient mice were generated previously and obtained from Dr. Tak W. Mak's laboratory. Details of the creation of the transgenic mouse have been previously characterized [72]. Briefly, exons 3–7 were excised and the first coding exon was modified to contain a premature codon. Absence of DJ-1 mRNA and protein was also documented in our studies below.

### 5.4 LPS 24 h intratracheal model

The intratracheal instillation was performed under sterile conditions. Animals were anesthetized with 3–5% isoflurane. The animal was positioned on a sloped surgical area. A small incision was made near the anterior aspect of the neck (throat region). The platysma and the anterior tracheal muscles were bluntly dissected to visualize and access the rings of the trachea. LPS (Escherichia Coli LPS 026:B6 and 055:B5, Sigma-Aldrich, St. Louis) was administered intratracheally (10 mg/kg) using a 30G needle in WT and DJ-1−/− mice in 50 μL volume. Equal volume of 1x PBS was administered in controls. All animals received fluid resuscitation with 50 ml/kg saline and 0.2 mg/kg Buprenex through subcutaneous injections twice daily. Animals were either sacrificed 24 h and assessed for various endpoints or continued for 7 days.

### 5.5 LPS 4 h ‘two hit’ mechanical ventilation model

WT and DJ-1−/− animals were randomized to (non-ventilated) spontaneous breathing (saline + NV), spontaneous breathing with LPS (LPS + NV), mechanical ventilation (saline + MV), or mechanical ventilation with LPS (LPS + MV). LPS was initially delivered (as before) using a 30G needle and allowed to recover for 5 min. The trachea was then cannulated (18-gauge steel cannula, BD Biosciences). The mice were placed on a custom built 6 mice pin apparatus which is connected to a ventilator (Servo 900c, Siemens, Sweden) for 4 h with the following settings: 110 breaths/min; PEEP = 2 cm H_2_O; I:E = 1:2; PIP = 11 cm H_2_O; O_2_ sat = 50%. Rectal temperatures were monitored in the duration of MV and maintained between 36.0 and 37.5 °C using a heating pad.

### 5.6 Pulmonary function tests

Twenty-four hours following LPS treatment, mice were anesthetized with ketamine and xylazine (i.p.), a neck midline incision was made to expose the trachea and facilitate intubation with an 18-gauge stainless steel cannula (BD Biosciences Canada, Mississauga, Ontario, Canada). The mice were then placed in a supine position and connected to the flexiVent system (SciReq Inc., Montreal, QC) for *in vivo*, ventilator-based assessment of respiratory mechanics [73]. Mice were ventilated at 150 breaths per minute, with a tidal volume of 10 mL/kg and a positive end expiratory pressure (PEEP) of 3 cm.H_2_O. Pressure–volume (PV) maneuvers were conducted to assess respiratory mechanics. Dynamic pressure volume curves were obtained by incrementally inflating lung to 30 cm.H_2_O and then reversing process while recording volumes. Area of the PV curve was calculated by the FlexiVent software. Respiratory tone was assessed using the linear first-order single compartment model, which uses forced oscillation to calculate resistance of the total respiratory system (Rrs), as well as compliance (C) and elastance (E). Distinction between airway and tissue mechanics was further assessed using constant phase model (CPM). All data points were collected using the flexiVent software and analyzed offline using Excel (Microsoft, Redmond, WA, USA) and Prism.

### 5.7 Tissue collection

Mice were sacrificed following 4 h or 24 h of respective procedures and tissues were harvested for analysis. Whole blood was collected by cardiac puncture, centrifuged, and serum separated for biochemical and mediator analysis. The right lung was formalin-fixed for histology and the left lung was snap-frozen and stored at −80 °C for protein and RNA analyses.

### 5.8 Bronchoalveolar lavage fluid (BALF)

Bronchoalveolar lavage was performed on a subset of animals. The trachea was cannulated (BD Biosciences, Canada) and instilled and withdrawn with three 0.5 mL aliquots of 1x PBS. The collected volume was spun down at 2500 g to collect cells for total and differential counting, and supernatant was stored at −80C for total protein and IgM analysis.

### 5.9 Histopathology and lung injury assessment

Lungs were fixed in 10% formalin and immersed in the same solution before tissue processing into paraffin-embedded blocks and sectioned onto slides in 6–8 μm sections. Slides were stained with hematoxylin and eosin (H&E) for routine histopathological assessment or extra unstained slides were used for future immunohistochemistry. For immunohistochemistry, tissue sections were deparaffinized by xylene incubation and hydrated with subsequent ethanol washes. Antigen retrieval was performed using hot citrate buffer (10 mM, pH 6). Slides were blocked with 10% BSA for 1 h, washed, and then incubated with primary antibody overnight at 4 °C. Endogenous peroxidase activity was blocked by incubating slides in 3% hydrogen peroxide before incubating with secondary antibody (anti-rabbit) for 1 h at room temperature. Slides were developed with DAB substrate for 15 min and counterstained with hematoxylin. Images were acquired on Olympus upright BX50 microscope. Lung injury scores were determined by a blinded pathologist using 10 random fields. Lung injury was based on assignment of a score of 0 (no injury), 1 (less than 25% involved), 2 (25–50% involved), or 3 (more than 50% involved) based on the presence of alveolar fibrin/edema, alveolar hemorrhage, septal thickening, and cellular infiltration. Lung injury scores were presented as a composite of the four criteria.

### 5.10 Ex vivo ROS production measurement

ROS production in the vasculature of the isolated perfused lung was assessed by real-time imaging of endothelial DCF fluorescence *in situ* as previously described [74] In brief, endothelial cells of isolated perfused murine lungs were loaded with H_2_DCF-DA (2 μMol/L) and DCF fluorescence in lung venular capillaries was imaged under a custom-built upright microscope equipped with a near-monochromatic light source (Polychrome V; TILL Photonics, Martinsried, Germany) at an excitation wavelength of 488 nm and an emission of >515 nm. ROS production in response to a bolus infusion of 1000 U/mL TNF-α or saline (control) was quantified as change in DCF fluorescence relative to baseline [75].

### 5.11 Levels of inflammatory mediators

Inflammatory marker levels in lung tissue lysates were measured using a custom murine specific Procarta Cytometric Bead Array (Affymetrix Panomics, Santa Clara, CA), according to manufacturer's instructions.

### 5.12 Propidium iodide staining

Following incubation with appropriate stimuli, cells were incubated with propidium iodide (50 μg/mL) and analyzed using a BD FACS Canto Flow Cytometer. Data were analyzed using BD FACS DIVA software. A minimum of 10,000 events were collected and analyzed at excitation wavelength of 488 nm and emission wavelength of 690 nm.

### 5.13 Annexin V staining

Cells were washed twice using 1x Phosphate Buffered Saline (PBS). Cells were resuspended in diluted 1X Binding Buffer (100 μl of each sample was taken and mixed with 99 μl of 1X Binding Buffer) (TREVIGEN, Cat # 4830-05-2, Lot # 015A7) and 1–5 μl of Annexin V FITC (Enzo, ALX-850-020-KI02). Cells were incubated at room temperature for 10 min, after which they were washed with 1X PBS. Cells were again resuspended in 200 μl of pre-diluted 1X Binding Buffer. Cells were then stained with propidium iodide (Sigma) (to measure late stage apoptosis. Cells were flicked and resuspended prior to analysis using a BD FACSCanto cytofluorometer with BD FACSDiva software version 5.0.1 (BD Biosciences, San Jose, CA).

### 5.14 Lactate dehydrogenase (LDH) levels

LDH levels in supernatant of Beas-2b cells and in BALF of mice were assessed using Cytotoxicity Detection Kit (LDH) (Cat. No. 11644793001, Roche Diagnostics).

### 5.15 Immunoprecipitation (IP)

0.5 mg or 1.0 mg cell or tissue lysates respectively were incubated with the primary antibody overnight on a rotating shaker. Following washing, the mixture was then incubated with A/G Agarose beads suspension (Santa Cruz or Pierce) for 1 h at room temperature. Following washing (3–5 times), the pellet was re-suspended in SDS loading buffer and boiled for 5 min. Supernatants were loaded onto gel and electrophoresis was performed and analyzed as detailed above.

### 5.16 Assessment of mRNA expression

Expression of mRNA levels of the various genes were evaluated using Real-time PCR, as previously described [71]. In brief, total RNA was extracted using Trizol (Ambion, Life Technologies) according to manufacturer's instructions. First Strand cDNA was synthesized with 2 μg of RNA samples for tissues and 1 μg of RNA samples for cells using the Superscript First strand synthesis system for RT-PCR (Invitrogen, Life technologies). Real-time PCR (qPCR) was performed with the ABI 7900HT Real Time PCR system (Applied Biosystems, Foster City, CA). Primers were generated and purchased from Primer Quest program (IDT DNA Technologies). Primer sequences are as follows: HMOX-1, *f* 5′ TAGCCCACTCCCTGTGTTTCCTTT3′, *r* 5′ TGCTGGTTTCAAAGTTCAGGCCAC 3′, GPX-1, *f* 5′AGCGCTAGTACGGATTCCACGTTT3′, *r* 5′ATTCTCAATGAGCAGCACCTTGCC3′, INOS, *f* 5′ CAAAAACTGGGGCAGTGG 3′, *r* 5′ CCACTCGGGCATCTGGTAG 3′, GAPDH, *f* 5′ AGAAACCTGCCAAGTATGATGACA 3′, *r* 5′ TGAAGTCGCAGGAGACAACCT 3′, FAS, *f* 5′TGTGGGCTACTGTGTTGCTT3′, *r* 5′TCGTTTGAGTTGTGGGCAGT3′ and NQO-1, *f* 5′GTCACGGCTATCCACAACCA3′, *r* 5′CGCTCGGCTAACAAACTCCT3’. The relative change in gene expression was calculated by the ΔΔCt method (Applied Biosystems) from triplicate determinations using GAPDH as a housekeeping gene.

### 5.17 Assessment of protein expression

Expression of proteins were assessed using Western Blots, as previously described [71]. In brief, snap frozen tissues were grounded using chilled mortal and pestle and a portion of the homogenized tissue was placed with lysis buffer. Cells were immediately ruptured in lysis buffer. Tissue & cell lysates were centrifuged at 15000 g for 10 min. A portion of the supernatant was used for determining the protein concentration using 90 the Bradford Assay. Lysates with equal amounts of protein were then mixed with 4x sample loading buffer and then separated on 8–15% SDS-polyacrylamide gels. They were subsequently transferred onto polyvinylidene difluoride (PVDF, Millipore) membrane. The membranes were blocked with 5% skim milk powder in Tris-buffered saline containing 0.1% Tween (TBS-T), probed with the following antibodies: rabbit monoclonal (DJ-1, HMOX-1, phospho-JNK, total JNK, Keap1, cleaved Caspase 3, total Caspase 3 at 1:100, Cell Signaling), mouse monoclonal (β-Actin, NOX2, p47phox, FAS at 1:200, Santa Cruz) or rabbit polyclonal (Nrf2, Ubr7, 1:1000, Abcam) in 10% BSA in TBS-T for an hour at room temperature or overnight at 4 °C. Following washing with TBS-T, they were incubated with horseradish peroxidase conjugated secondary antibodies for an hour, there after developed with enhanced chemiluminescence (Amersham Pharmacia Biotech) and visualized on film using Bio-Rad Gel Doc 2000. Densitometry was performed using GelQuantNet software.

### 5.18 Statistical analyses

Mice were randomized to treatment groups and investigators were blinded to genotype. Observers assessing endpoints were blinded to genotype and group assignment. Survival studies were analyzed using Log Rank (Mantel-Cox) tests. Unless otherwise stated, data are presented as mean±standard error of mean (SEM). Differences between groups were determined using Mann-Whitney, or two-way ANOVA followed by Bonferroni post-hoc test to account for both “genotype” and “treatment”. The statistical package GraphPad PRISM 6.0 was used for analysis.

## Sources of funding

Supported by the 10.13039/501100000024Canadian Institutes of Health Research (MOP-130331 and OCN 126573; C·C.d.S.), the Early Research Award from the Ministry of Research and Innovation (ER 10-07-182; C·C.d.S.), the Ontario Graduate Scholarship (H.A.), and St. Michael's Hospital Li Ka Shing Knowledge Institute Graduate Scholarship (H.A.). The funders had no role in study design, data collection and analysis, decision to publish, or preparation of the manuscript.

## Author contributions

Conception and design, C·C.d.S, and J.C.M. Data collection, analysis and interpretation, H.A, Y·S, T.M.-G, P.R.M.R, A.K·V, A.P.T.M, X.H, A.T, W.M.K, S.G, J.N.T, and H.Z. Drafting the manuscript for important intellectual content, H.A, A.V·K, A.P.T.M, S.G, and J.N.T. Critical revision of the article, H.A, A.V·K, A.P.T.M, S.G, J.N.T, and C·C.d.S.

## Declaration of competing interest

None.

## List of Abbreviations

ALI: acute lung injury
ARDS: acute respiratory distress syndrome
ATF3: activating transcription factor 3
BMM: bone marrow macrophage
BALF: bronchoalveolar lavage fluid
Beas-2b: human bronchial epithelial cell line
C: compliance
CPM: constant phase model
CST: quasistatic compliance
DA: diffuse alveolar damage
Keap-1: Kelch Like ECH Associated Protein 1
E: elastance
Gpx1: Glutathione Peroxidase 1
H: peripheral tissue dampening
Hmox-1: heme oxygenase 1
HPMEC: Human pulmonary microvascular endothelial cells
LDH: lactate dehydrogenase
IL: interleukin
KC: keratinocyte chemoattractant
LPS: lipopolysaccharide
JNK: *c*-Jun *N*-terminal kinase
MCP-1: monocyte chemoattractant protein-1
MIP-1: macrophage inflammatory protein 1
MV: mechanical ventilation
Nqo1: NAD(P)H Quinone Dehydrogenase 1
Nrf2: Nuclear Factor, Erythroid 2 Like 2
PARK7: Parkinson Disease (Autosomal Recessive, Early Onset) 7
NADPH: nicotinamide adenine dinucleotide phosphate
NLRP3: NOD-, LRR- and pyrin domain-containing protein 3
NOX2: NADPH oxidase 2
PEEP: positive end expiratory pressure
PI: propidium iodide
PV: pressure volume
R_N_: Newtonian resistance
ROS: reactive oxygen species
Rrs: total respiratory system
TLR: toll like receptor
TNF: tumor necrosis factor
siRNA: short interfering RNA
VILI: ventilator-induced lung injury
UTR: untranslated region
WT: wild type

## Appendix A Supplementary data

Application Application
