# Supplementary material for: Protective function of DJ-1/PARK7 in lipopolysaccharide and ventilator-induced acute lung injury
Source: Redox Biol. 2020 Nov 17;38:101796. doi: 10.1016/j.redox.2020.101796 (PMC7695876; doi:10.1016/j.redox.2020.101796)
Supplement: Application [file mmc1.docx]

**Supplementary Figure Legends**

**Supplementary Figure 1: Absence of DJ-1 exacerbates LPS-induced acute lung injury. A)** Pressure volume curves (n=5-8 per group) of WT and DJ-1-/- mice obtained using SciReq FlexiVent 24 hours post saline (SAL) or LPS instillation. Points represent volume means ± SEM recorded at given pressure. **B)** Resistance (Rrs) and **C)** compliance (C) of WT and DJ-1-/- mice obtained using SciReq FlexiVent 24 hours post saline or LPS instillation. **D)** Representative gross morphology of lung following Evans Blue injections in WT and DJ-1-/- mice treated with LPS. **E)** BALF IgM (n=8-13) in WT and DJ-1-/- mice at 24 hours after saline and LPS treatment. Bar graphs represent means ± SEM of n=5-8 mice per group (*<0.05, **<0.01, Two-way ANOVA analysis).

**Supplementary Figure 2: Absence of DJ-1 exacerbates lung injury in a two-hit model. A)** Representative H & E images of WT and DJ-1-/- lungs at 4 hours after saline or LPS instillation with or without MV. Pulmonary **B)** Resistance (Rrs) and **C)** compliance (C) of WT and DJ-1-/- mice obtained using SciReq FlexiVent at 4 hours post saline or LPS instillation. **D)** Blood PaO_2_:FiO_2_ ratio and **E)** pH levels in WT and DJ-1-/- animals at 4 hours post saline or LPS instillation (n=5-7 for saline, 8-11 for LPS). Bar graphs represent means ± SEM (*p<0.05, **p<0.01, two-way ANOVA analysis).
