# Supplementary figures and images for: Protective function of DJ-1/PARK7 in lipopolysaccharide and ventilator-induced acute lung injury
Source: Redox Biol. 2020 Nov 17;38:101796. doi: 10.1016/j.redox.2020.101796 (PMC7695876; doi:10.1016/j.redox.2020.101796)

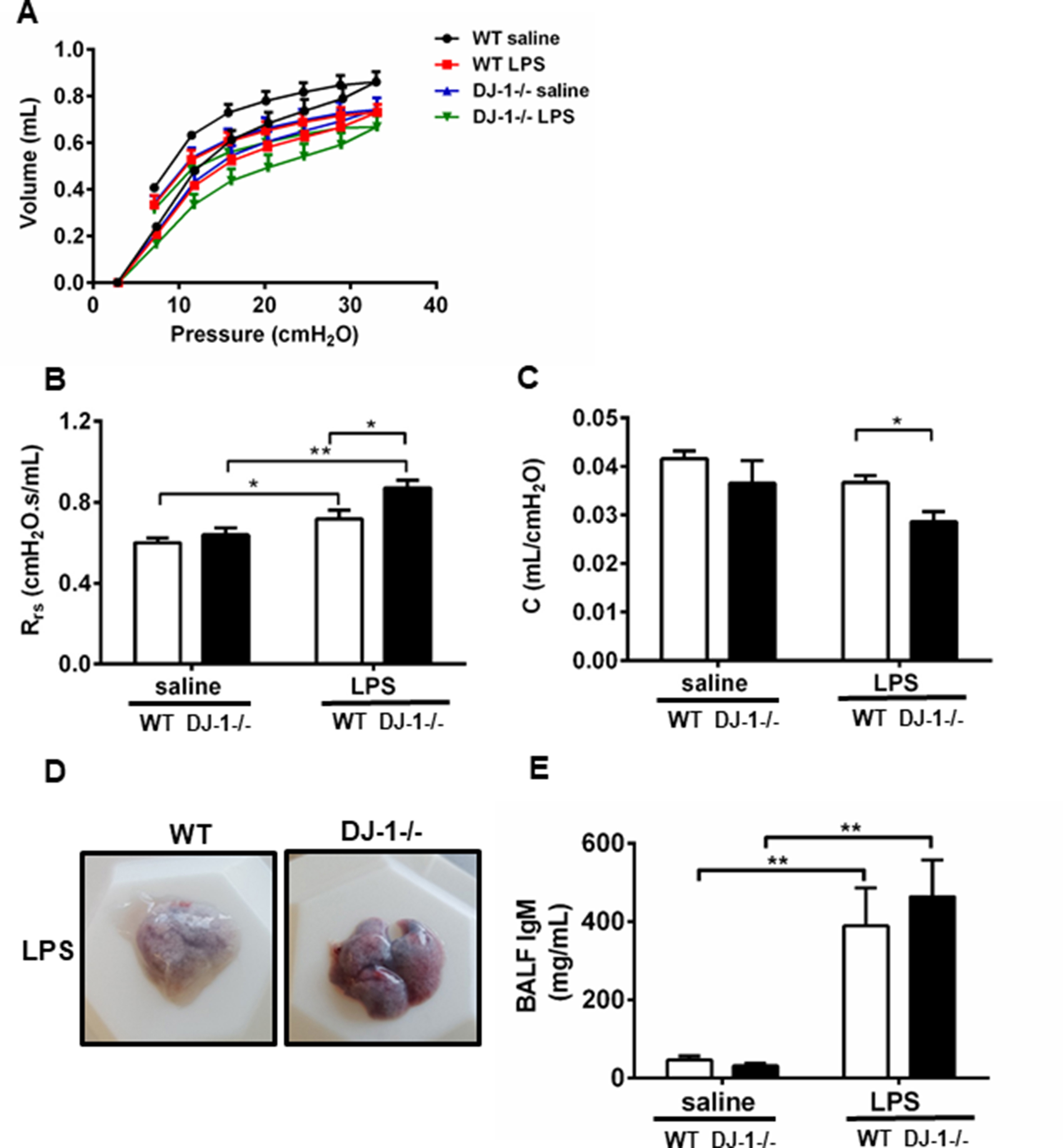

Supplement: Supplementary file 2 [file mmcfigs1.jpg]

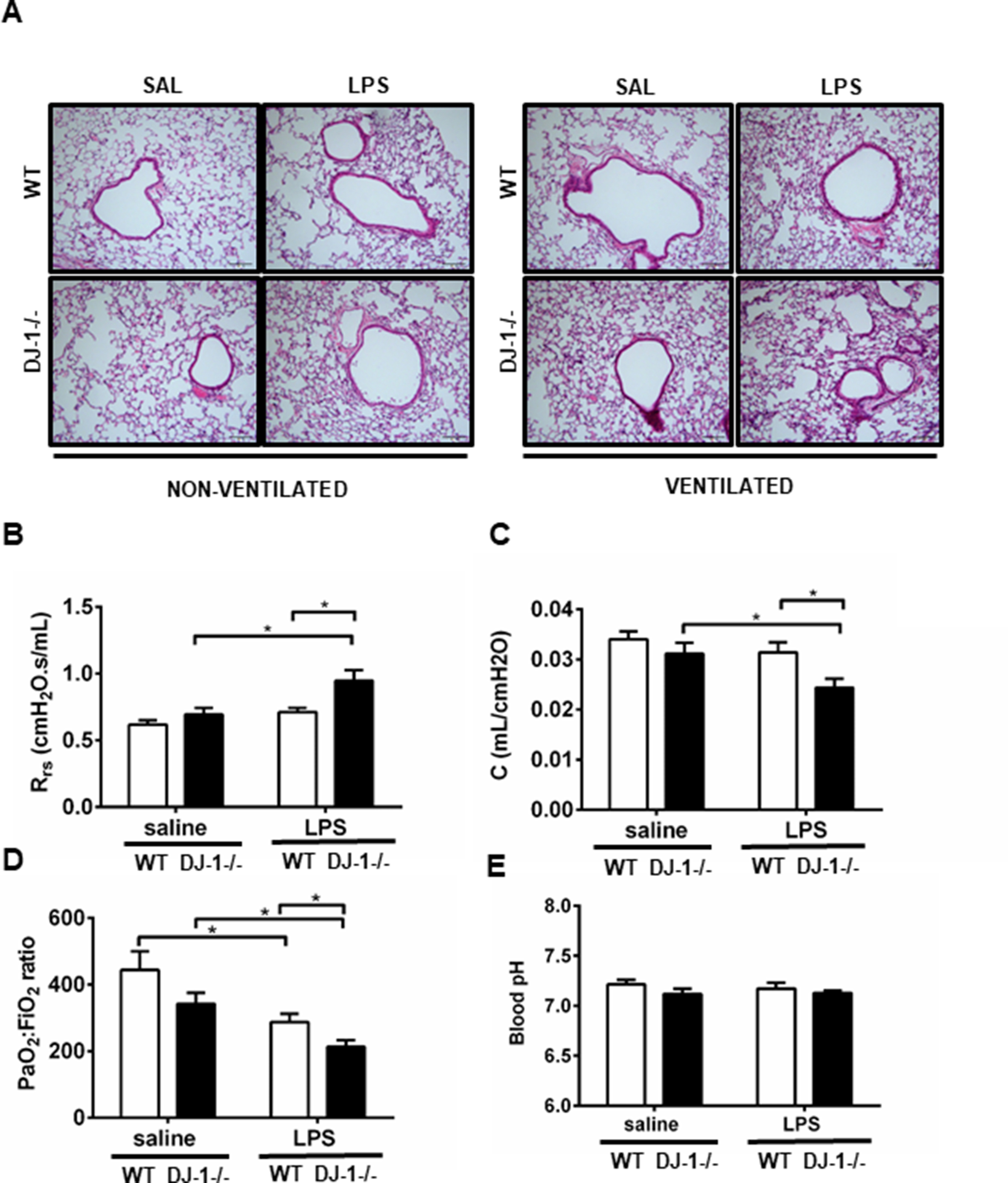

Supplement: Supplementary file 3 [file mmcfigs2.jpg]
